# Unraveling the enigma: housekeeping gene *Ugt1a7c* as a universal biomarker for microglia

**DOI:** 10.3389/fpsyt.2024.1364201

**Published:** 2024-04-11

**Authors:** Wonju Kim, Minji Kim, Beomsue Kim

**Affiliations:** Neural Circuit Research Group, Korea Brain Research Institute, Daegu, Republic of Korea

**Keywords:** housekeeping gene, microglial cell, biomarker, UGT1A7C protein, mouse, embryo development, transcriptome analysis, neurologic disorders

## Abstract

**Background:**

Microglia, brain resident macrophages, play multiple roles in maintaining homeostasis, including immunity, surveillance, and protecting the central nervous system through their distinct activation processes. Identifying all types of microglia-driven populations is crucial due to the presence of various phenotypes that differ based on developmental stages or activation states. During embryonic development, the E8.5 yolk sac contains erythromyeloid progenitors that go through different growth phases, eventually resulting in the formation of microglia. In addition, microglia are present in neurological diseases as a diverse population. So far, no individual biomarker for microglia has been discovered that can accurately identify and monitor their development and attributes.

**Summary:**

Here, we highlight the newly defined biomarker of mouse microglia, UGT1A7C, which exhibits superior stability in expression during microglia development and activation compared to other known microglia biomarkers. The UGT1A7C sensing chemical probe labels all microglia in the 3xTG AD mouse model. The expression of *Ugt1a7c* is stable during development, with only a 4-fold variation, while other microglia biomarkers, such as *Csf1r* and *Cx3cr1*, exhibit at least a 10-fold difference. The UGT1A7C expression remains constant throughout its lifespan. In addition, the expression and activity of UGT1A7C are the same in response to different types of inflammatory activators’ treatment *in vitro*.

**Conclusion:**

We propose employing UGT1A7C as the representative biomarker for microglia, irrespective of their developmental state, age, or activation status. Using UGT1A7C can reduce the requirement for using multiple biomarkers, enhance the precision of microglia analysis, and even be utilized as a standard for gene/protein expression.

## Introduction

**Figure 1:**
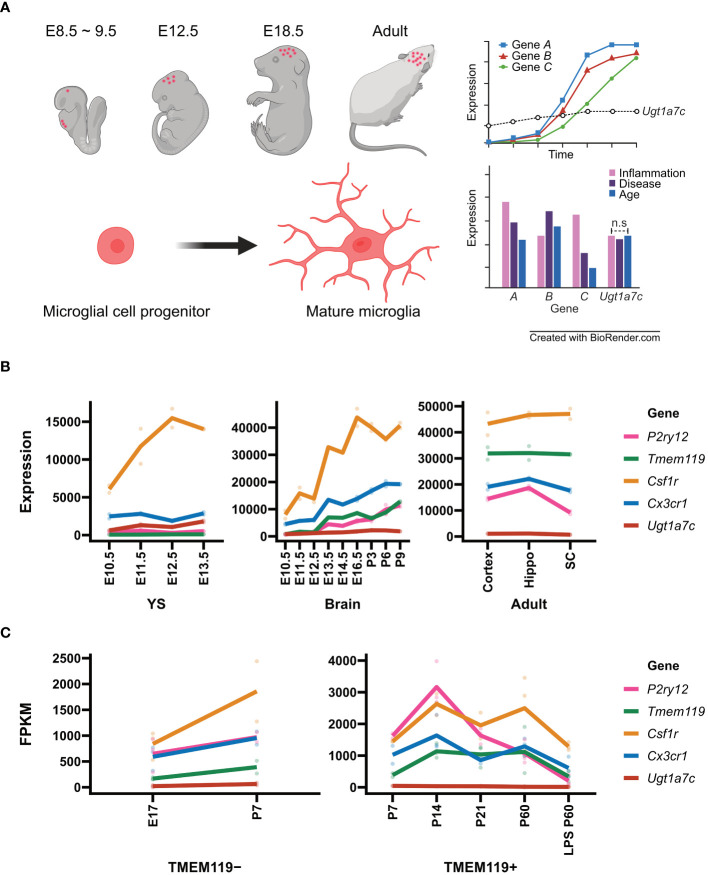
Microglial marker genes and *Ugt1a7c*. **(A)** Schematic diagram of microglial housekeeping gene. **(B)** Line plot of expression of microglial marker genes and *Ugt1a7c* across the microglial development including yolk sac (YS), pre-mature brain (Brain), and mature brain (Adult). From GSE79812, expression profiling data of microglial marker genes and *Ugt1a7c* were acquired. Units on the y axes are arbitrary. **(C)** Line plot of expression of microglial marker genes depending on TMEM119 protein expression. Data were acquired from NCBI BioProject (Accession PRJNA307271). For microglial marker genes, *Csf1r*, *Cx3cr1*, *P2ry12*, and *Tmem119* were selected. A line represents the mean value of replications (colored spots). E, embryonic day; P, postnatal day; Hippo, hippocampus; SC, spinal cord; LPS, lipopolysaccharide. Created with BioRender.com.

Microglia are involved in immune responses as tissue-resident macrophages of the central nervous system (CNS) (1–3). In addition to the immune role, their cellular activities are involved in the neuronal array regarding the refinement of synaptic connections and the elaboration of neuromodulatory factors for cognitive ability (4–7). They take up approximately 5 ~ 12% of the total cells in the mouse brain, with a diversity of transcriptional module combinations and levels of crowd across the brain region (8–12). Given their functional role and prevalence in the brain, microglial regulation has a high potential to develop brain disease therapy (13–15). Indeed, recent studies have linked microglia to neurodevelopmental and psychiatric diseases and neurodegenerative diseases (16–19). Accumulated mouse *in vivo* lineage tracing results indicate that microglia at different developmental stages are characterized by unique molecular features (20–23). Erythromyeloid progenitors (CD45^-^c-Kit^+^) arise before the end of embryonic day (E) 8 during the first wave of hematopoiesis in the yolk sac (24). Erythromyeloid progenitors - derived primitive macrophage progenitors (CD45^+^c-Kit^lo^CX3CR1^-^) colonize the developing brain at E9.5 and further differentiate into microglia in a Myb-independent manner via the PU.1- and IRF8-dependent pathway (22, 25). Regardless of distinctive ontogeny, microglia also express general macrophage markers such as CD11b, CSF1-1 receptor CD115, surface glycoprotein F4/80, and fractalkine receptor CX3 chemokine receptor CX3CR1 (24, 26–28). Although the expression level is not very high, microglia even express the hematopoietic marker CD45 (24, 29). However, most of the general markers does not satisfy the requirements for covering all stages of microglia. F4/80 is present from E9.5, but its expression is undetectable at E8.5 in the brain according to fate-mapping analysis of CSF1R (CD115)-expressing cells (30). CX3CR1 is expressed in the gut region at E8.5 and is sparsely visualized throughout the embryo. At stage E8.5 to 9.0, it was detectable in neural tissue within telencephalic vesicle. At E9.5, CX3CR1 microglial precursor cells were detected in the surface ectoderm [(31), **Figure 1A**]. As a result of these restricted generalities, it is common for researchers to utilize at least two biomarkers in lineage tracing experiments [(32), **Table 1**]. However, this approach often results fragmented data and unclear interpretation during specific time periods.

**Table 1:** Microglia marker.

Gene	Protein	UniProt	Synonyms	Functions	JAX ID	References
Adgre1	Adhesion G protein-coupled receptor E1	Q61549	Cell surface glycoprotein F4/80, EGF-like module receptor 1	Involved in cell adhesion and probably in cell-cell interactions specifically involving cells of the immune system; Play a role in regulatory T-cells development	038175	(12, 33–35)
Adora3	Adenosine A3 receptor	Q61618	A3AR, Gpcr2	Potential targets for acute or chronic pain management	066730	(36–40)
Aif1	Allograft inflammatory factor 1	O70200	Iba1, Ionized calcium-binding adapter molecule 1	Enhances the actin-bundling activity of LCP1; Plays a role in RAC signaling and in phagocytosis		(41)
Cd14	Monocyte differentiation antigen CD14	P10810	Myeloid cell-specific leucine-rich glycoprotein, CD14	Coreceptor for bacterial lipopolysaccharide (LPS); Mediating the innate immune response to the bacterial LPS	003726	(42–44)
Cd80	T-lymphocyte activation antigen CD80	Q00609	Activation B7-1 antigen (B7), CD80	Involved in the costimulatory signal essential for T lymphocytes activation	036705	(45–47)
Csf1r	Macrophage colony-stimulating factor 1 receptor	P09581	CSF-1 receptor, CD115	Acts as cell-surface receptor for CSF1 and IL34 and plays an essential role in the regulation of survival, proliferation and differentiation of hematopoietic precursor cells, especially mononuclear phagocytes, such as macrophages and monocytes; Play a role in the development of microglia macrophages	021212	(48, 49)
Cst3	Cystain-C	P21460	Cystain-3	Related cerebral amyloid angiopathy; cysteine protease inhibitor; regulate lysosomal enzyme activity		(39, 50)
Cx3cr1	CX3C chemokine receptor 1	Q9Z0D9	Fractalkine receptor, C-X3-CCKR-1	CX3CR1-CX3CL1 signaling exerts distinct functions in different tissue compartments, such as immune response, inflammation, cell adhesion and chemotaxis	005582	(26, 51)
Ecscr	Endothelial cell-specific chemotaxis regulator	Q3TZW0	ECSM2, ARIA	Role in angiogenesis and apoptosis via modulation of the actin cytoskeleton		(52)
Entpd1	Ectonuceloside Triphosphate Diphosphohydrolase 1	P55772	NTPDase-1, CD39	Hydrolyze ATP and other nucleotides to regulate purinergic neurotransmission; Involved in microglial senses and suppresses neuronal hyperexcitability in epilepsy		(53)
Fcgr1	High affinity immunoglobulin gamma Fc receptor I	P26151	Fc-gamma RI (FcRI), CD64	Functions in both innate and adaptive immune responses		(54–56)
Hexb	Beta-hexosminidase subunit beta	P20060	N-acetyl-beta-glucosaminidase subunit beta	Hydrolyzes the non-reducing end N-acetyl-D-hexosamine; responsible for the degradation of GM2 gangliosides in the presence of GM2A	030864	(57, 58)
Itgam	Integrin alpha-M	P05555	Cell surface glycoprotein MAC-1 subunit alpha, CD11b	Implicated in adhesive interaction of monocytes, macrophages, and granulocytes; Mediating uptake of complement-coated particles	005515	(59–61)
Mef2a	Myocyte-specific enhancer factor 2A	Q60929	RSRFC4, Serum Response Factor-Like Protein 1	Regulating autophagy related genes; Maintenance of microglia homeostasis	010587	(62–64)
P2ry12	P2Y purinoceptor 12	Q9CPV9	P2Y12	Inhibit the adenylyl cyclase second messenger system; Required for normal platelet aggregation and blood coagulation	034727	(65–67)
P2ry13	P2Y purinoceptor 13	Q9D8I2	P2Y13, Gpr86	Regulate microglial morphology, surveillance, and resting levels of interleukin 1β release		(68)
Ptprc	Receptor-type tyrosine-protein phosphatase C	P06800	Leukocyte common antigen (L-CA), CD45	Acts as a positive regulator of T-cell coactivation upon binding to DPP4	002014	(69, 70)
Sall1	Sal-like protein 1	Q9ER74	Zinc finger protein Spalt-3	Critical regulator of organogenesis and microglia identity; Associated Townes–Brock syndrome	033318	(71–74)
Sall3	Sal-like protein 3	Q62255	Spalt-like protein 3	Mutations of these gene are associated with congenital disorders in human. Binding DNMT3A reduces DNMT3A-meidated CpG island methylation		(73)
Sparc	SPARC	P07214	BM-40	Regulate cell growth through interactions with the extracellular matrix and cytokines; Regulates the distribution and branching of mature microglia	003728	(75, 76)
Spi1	Transcription factor PU.1	P17433	PU.1, SFFV proviral integration 1 protein	Plays a crucial role in determining macrophage lineages and microglial genesis and is a major factor in selecting the set of enhancers expressed by microglia	006147	(71, 77, 78)
Tgfa	Proptransforming growth factor alpha	P48030	TGF-Alpha	Promote anchorage-independent cell proliferation; Regulate the pathogenic activities of astrocytes		(79, 80)
Tmem119	Transmembrane protein 119	Q8R138	Osteoblast induction factor (OBIF)	Promotes the differentiation of myoblasts into osteoblasts	031823	(65, 81, 82)
Ugt1a7c	UDP-glucuronosyltransferase 1A7	Q6ZQM8		UDP-glucuronosyltransferase (UGT) that catalyzes phase II biotransformation reactions in which lipophilic substrates are conjugated with glucuronic acid to increase the metabolite’s water solubility, thereby facilitating excretion into either the urine or bile		(83)

Inspired Jurga and colleagues’ work (32), data were acquired from www.uniprot.org and www.jax.org.

Since the publication of the fate mapping study in 2010, there has been a significant increase in information regarding microglia’s ontogeny, maintenance, neuroimmune activities, and interactions (20). Microglia express heterogeneous profiles with different shapes, gene expression patterns, and even function (84, 85). The integration of several microglia biomarkers for translational/transcriptomic analysis is an informative feature based on information about microglia (84, 86–90). A single microglia biomarker was not sufficient to identify them and track their ontogeny and characteristics, especially when there was an interaction with other types of glia/neurons and a transition of status (13, 91–93). For example, P2RY12 downregulation occurs upon microglia activation (65). TMEM119 appears to be influenced by inflammatory responses and environmental factors including TGF-β and LPS (65, 81, 94). Sall1 expression is highly correlated with TGF-β1 signaling and varies between microglial cell lines (71, 72). The activation state of microglia also strongly impacts the expression of CD11b (59–61), CD115 (48, 49) and F4/80 (12, 33–35). Identifying a novel biomarker for monitoring microglia remains a major challenge in microglial biology.

Many aspects of microglial development and origin have been clarified through fate mapping studies in mice (20, 95). Depletion–repopulation experiments have demonstrated that microglia rely solely on self-renewal and are not influenced by other organs (96). Microglia were once considered to be uniform cells that respond to their environment because of their distinctive feature: “single origin” and “self-renewal.” However, recent research indicates that microglia exhibit high diversity in terms of morphology, function, and gene expression (27, 57, 71, 97–100). This heterogeneity in microglia is due to various factors, including intrinsic factors such as species, gender, and genetic background, and extrinsic factors such as pathogens, nutrition, and microbiota (10, 80, 101–103). The conventional *in vitro*-based classification distinguished “M1” and “M2” microglia, with M1 indicating neurotoxic and proinflammatory microglia and M2 representing neuroprotective and anti-inflammatory microglia (104–107). Nonetheless, the dichotomous classification has been replaced by multiple subclass-cluster classification based on transcript combinations and surface protein combinations with the advent of technologies such as single cell RNA seq and single cell mass spectrometry (CyTOF) (98, 108–115).

Low gene expression is often disregarded, as it is anticipated to have a negligible impact on cells. A recent study discovered a new microglial biomarker, UDP-glucuronosyltransferase 1a7c (UGT1A7C). The discovery was made during the analysis of the target gene to a microglia-specific BODIPY-based fluorescent dye called CDr20 (83). Thanks to the low yet sufficient level of the enriched UGT1A7C enzyme, the microglia population efficiently converts the small chemical into a fluorescent active form (57). The development of high-performance fluorogenic chemical probes enables the visualization of microglia with the biomarker and allows UGT1A7C to enter the microglia research area with its unique approaching capacity that can act both *in vitro* and *in vivo* (116). From this perspective, we present a concise overview of microglia and UGT1A7C, while also shedding light on areas that faced technical limitations and did not receive adequate attention due to previous doubts about their existence in the brain.

## Discussion

Microglia originate from the yolk sac and go through different phases, such as erythromyeloid and macrophage precursors. Once they enter the brain, microglia take on the role of tissue-resident macrophages and become involved in neuromodulation, which explains the diverse protein expression patterns observed in different developmental stages and microglia functions. To track microglia, different markers that match their specific property of interest are utilized. As technology advances, the list of attributes specific to each stage of microglia, including the genes they express, is continuously growing. Consequently, microglia are undergoing additional subclassification according to their recognized features.

### Microglia heterogeneity

Microglia exhibit variations across distinct brain regions. Microglia in the prefrontal cortex (PFC) express high levels of *Cx3cr1*, *P2ry12*, and *Tmem119* and low levels of *Apoe*, *Lyz2*, and *Spp1*, which are involved in synaptic modulation and plasticity for learning and memory and in inflammation and immune response, respectively (117). Microglia in the striatum express significant amounts of *Map1b*, *Map2*, and *Tubb2a* and low levels of *Cd68*, *Lgals3*, and *Mrc1*, which are involved in cell movement and shape and for the suppressed activity for phagocytosis and lysosomal function against pathogens and injuries, respectively (117). Microglia in the midbrain express high levels of *Ccl2*, *Ccl5*, and *Il1b* and low levels of *Cx3cr1*, *P2ry12*, and *Tmem119*, caused by their active inflammation and immune response and low synaptic modulation and plasticity activities (117). Microglia in the cerebellum express high levels of *Gpx1*, *Gpx4*, and *Sod2* and low levels of *Cx3cr1*, *P2ry12*, *Tmem119*, and *Aif1*, indicating that they are sensitive to oxidative stress and metabolism, but show less function related to synaptic modulation and identity (118).

In addition to regional differences, the specific microenvironment is also linked to the molecular signature of microglia. For example, microglia actively express the fractalkine receptor (CX3CR1) when in contact with neurons that express its ligand, called fractalkine (CX3CL1) (26, 119–121). The contacted microglia are then actively involved in neural plasticity by pruning excess and/or weak synapses through the receptor-specific signaling pathway (122, 123). Another example is the interaction between the microglial TREM2 receptor and the neuronal ApoE ligand (124–126). The interaction plays a role in regulating microglial phagocytosis and inflammation as well as neuronal lipid metabolism and function (127–130). Lastly, Brain-derived neurotrophic factor (BDNF) secreted from neurons binds to TrkB receptors in microglia, enhancing neuronal survival and function (131–133). These examples collectively indicate that the microglial signature genes are largely controlled by the activity of neurons and other glia such as astrocytes, meaning that most of the signature genes are highly and temporally regulated by the microenvironment status of the brain (18, 77, 84, 134).

Using a single microglial biomarker in diversity studies is insufficient for classifying microglia (135, 136). A new trend has arisen where functional studies are using multiple microglial biomarkers to define different “subtypes” of microglia (136). However, it is frustrating to use more than two or three biomarkers to distinguish a microglial population from other glia/neurons. The development of a tool capable of continuous detection of all types of microglial population in any of the brain regions is necessary for future microglial studies regarding its cellular function in any “phenotype” of microglia appearing in a region and a condition impacted by external factors (117, 118). Therefore, it is crucial to ascertain the count of microglial subtypes, comprehend their localization, and identify the adjacent cells in every brain region while determining these biomarkers. Brain region-specific cellular relationships are currently characterized across the human brain as well as the mouse’s through single cell RNA sequencing with anatomical dissections (111, 137–139). Siletti and colleagues comprehensively analyzed 105 anatomical dissections of 4 human brains across 10 brain regions, including the cerebral cortex, hippocampus, cerebral nuclei, hypothalamus, thalamus, midbrain, pons, cerebellum, medulla, and spinal cord. Each single cell was classified into 31 superclusters and 461 clusters using a hierarchical classification system. According to this dataset, microglia were classified as a single supercluster and subsequently divided into 9 clusters (111). In contrast, other glial cell types such as astrocytes were classified as superclusters, comprising 13 clusters. Compared with glia, neurons showed significant variation across different brain regions. For example, medium spiny neurons (MSNs) were classified into 32 clusters, including eccentric MSN clusters (111). This study compares cell clustering combinations in adjacent anatomical dissections and offers new insights into the impact of cell type diversity on regional-specific variations across the human brain through single cell-level transcriptome analysis of regions. Comparative analysis of neighboring anatomical dissections reveals that when a new neural circuit is established for a particular function, gradual changes occur in the neuron’s surroundings, including microglia and astrocytes, instead of creating a “primary function neuron” all at once. Subsequently, the existing “primary function neuron” undergoes fine-tuning to transform into a new “primary function neuron” through interactions with its environment (111, 137). This means that not only individual cell function, but also the function of individual brain regions is strongly influenced by interactions between cells. As a result, identifying genes that are expressed regardless of regional differences and cell-cell interactions becomes a priority.

The gene-based heterogeneity of microglia, however, is limited because current knowledge is provided by information only about a specific snapshot in time. The diversity of microglia due to environmental factors, such as cell–cell interactions and disease states, is more precisely delineated when observed over a broad period. To track environmentally responsive microglial phenotypes and associated protein functions over time, reliable stable markers that are constitutively expressed and independent of the environment are essential. We serendipitously discovered UGT1A7C as a new type of microglia biomarker during the development of a fluorogenic microglia probe called CDr20, an enzyme that marks microglia regardless of environmental differences.

### UGT1A7C as a house-keeping biomarker of microglia

According to Matcovitch-Natan et al. RNA-seq data, the development of microglia was identified in three stages: early (until embryonic day 14), pre- (within a few weeks after birth), and adult microglia. They revealed a stepwise developmental program of microglia that is synchronized with the development of the brain. Early microglia were initially formatted with genes related to cell cycling and differentiation, such as *Mcm5* and *Dab2*. Thereafter, the expression of genes related to neurodevelopment, such as *Csf1* and *Cxcr2*, increased and reached its peak a few days before birth. *Cd14* and *Pmepa1*, which are representative genes for mature microglia, were found to be expressed primarily in adult microglia (62). In this dataset, we examined the expression patterns of well-known microglial markers including *P2ry12*, *Tmem119*, *Csf1r* and *Cx3cr1* compared to *Ugt1a7c* across different developmental stages (**Figures 1B, C**).

The gene *P2ry12*, which encodes P2RY12, is expressed approximately 82 times more in the adult stage than in the yolk sac stage [(62), **Figure 1B**]. P2RY12, which was initially identified on platelets as a mediator of platelet activation and blood clotting, is a G_i/o_-coupled purinergic receptor expressed in the CNS specifically by homeostatic microglia (140–144). Activation of P2RY12 through ATP/ADP induces rapid microglial chemotaxis and directional branching of microglial processes (65, 145). Additionally, it is involved in activities such as substrate-dependent cell migration and extension *in vitro* and *ex vivo*, as well as the regulation of microglial migration (65, 146, 147). P2RY12 is highly expressed along the microglial membrane under normal circumstances (57).. However, after an injury, P2RY12 downregulation occurs (65, 145). This means that microglia may promptly identify alterations in brain homeostasis and react appropriately by expressing P2RY12 (57, 65, 148, 149). The gene encoding transmembrane protein 119 (TMEM119) was expressed 803 times higher in the adult stage than in the yolk sac stage (62). *Tmem119*, also referred to as *Obif* (Osteoblast induction factor), is expressed exclusively in microglia in the brains of mice and humans, allowing for distinction from infiltrating blood-derived macrophages (81, 89). Its function in microglia remains unclear (81, 82, 89, 94). TMEM119 is located in the endoplasmic reticulum and plasma membrane (150–152). It is also expressed in several organs like the alimentary system, genitourinary system, limb, and skeleton as well as the brain (153, 154). A previous study found that frozen tissue samples of the frontal cortex from patients with Alzheimer’s had increased levels of *TMEM119* mRNA (81, 155). Transforming growth factor beta (TGF-β) induced an upregulation of *Tmem119* gene expression in cultured mouse microglia (156). In contrast, lipopolysaccharide (LPS), interleukin 4 (IL–4), or interferon-gamma (IFN-γ) induced downregulation of *TMEM119* gene expression in cultured human microglia (81, 89). The diverse expression patterns of TMEM119 appear to be influenced by inflammatory responses and environmental factors (82).

In contrast to the wide variation observed in the expression of *P2ry12* and *Tmem119*, the expression of *Csf1r* and *Cx3cr1* remains relatively stable across microglial developmental stages, with only 9-fold and 13-fold changes, respectively [(62), **Figure 1B**]. *Csf1r* encodes the colony-stimulating factor 1 receptor (CSF1R), a member of the tyrosine kinase receptor family (157). Upon stimulation by its ligands, including CSF1 and interleukin 34, CSF1R undergoes autophosphorylation of tyrosine residues in the intracellular domain, followed by activation of downstream signaling pathways (158). CSF1R is primarily expressed in the microglia of the brain and is crucial for their survival, proliferation, and differentiation (159–162). The gene *Cx3cr1* encodes the receptor for the C-X3-C chemokine fractalkine (CX3CL1), which is found in numerous leukocyte cells during early development (163–165). Signaling through CX3CR1-CX3CL1 exerts distinct functions in various tissue compartments, including immune response, inflammation, cell adhesion, and chemotaxis (163, 166–169). It controls the inflammation process that triggers atherogenesis, by facilitating the recruitment of macrophages and monocytes to inflamed atherosclerotic plaques, thus promoting cell survival (170, 171). CX3CR1 plays a crucial role in regulating the inflammatory response and synapse maturation in CNS microglia (122, 172, 173). During postnatal brain development, the brain participates in synaptic pruning, a natural process in which brain microglia eliminate extra synapses (122, 123, 133, 174, 175). Interestingly, despite the relative stability of both *Csf1r* and *Cx3cr1* expression, the fluctuation of their expression levels around 10-fold makes the two genes more suitable for distinguishing microglial subtypes rather than serving as housekeeping biomarkers for microglia (62).

Surprisingly, *Ugt1a7c* expression remains remarkably stable throughout the yolk sac, pre-microglia, and adult microglia stage, exhibiting only a 4-fold difference between maximum and minimum expression [(62), **Figure 1B**]. The peak expression period occurs between 3 and 6 days after birth, with expression levels remaining moderate thereafter as the microglia reach full maturity (62). The *Ugt1a7c* gene belongs to the UGT (UDP-Glycosyltransferase) gene family and is the only member enriched in microglia (176–179). The UGT gene families found in animals, plants, fungi, and bacteria facilitate phase II biotransformation reactions (180–184). They conjugate lipophilic substrates with glucuronic acid, from the UDP-glucuronic acid to the functional hydroxyl group of substrates, resulting in increasing hydrophilicity and facilitating excretion through bile and urine in the systemic organs eventually (176, 184–188). The role of *Ugt1a7c* in the brain, however, has not been extensively studied because its expression is not high compared with that of microglia-specific genes such as *P2ry12* and *Tmem119*, although its expression remains constant throughout development (**Figures 1B, C**).

**Figure 2:**
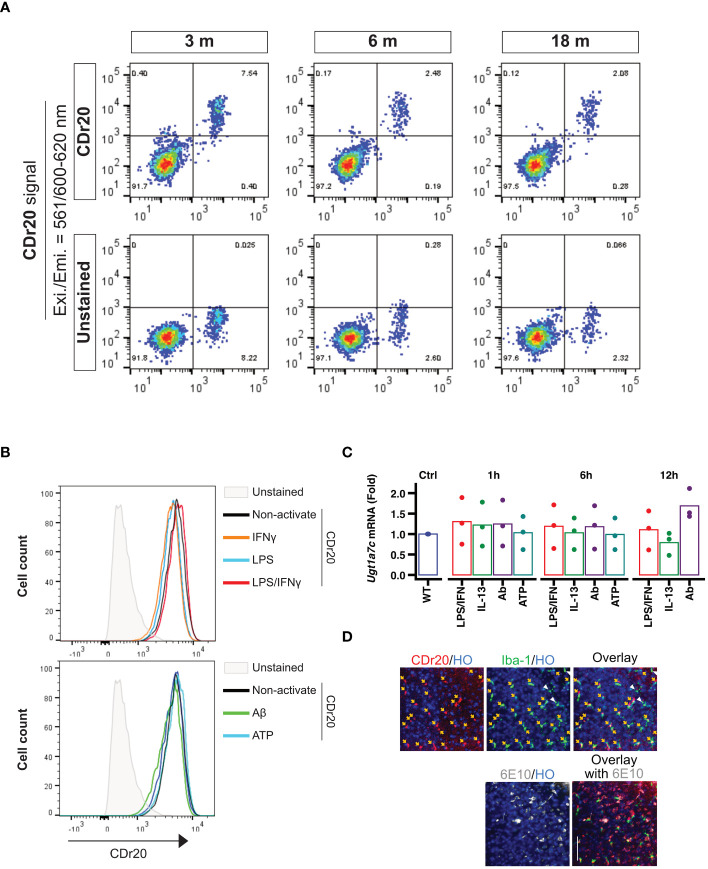
Environmental factor independent UGT1A7C. **(A)** Age-independent *Ugt1a7c*. The intensities from CDr20 (UGT1A7C-specific fluorescence substrate)-derived fluorescence were analyzed by treatment of live single cells dissociated from each age of the whole brain of a mouse. **(B)** Activation-independent *Ugt1a7c*. CDr20 fluorescence intensity was analyzed by each activation stimulation. **(C)** Activation-independent *Ugt1a7c*. *Ugt1a7c* mRNA expression levels were tracked at each time point. **(D)** AD-independent *Ugt1a7c*. Fluorescence image of the CDr20 (upper left, UGT1A7C-positive cells) in a live cortical brain slice after 30 minutes of treatment and of the immunostaining of Iba-1 (upper middle, microglia) and 6E10 (lower left, Aβ aggregates) after fixation of the tissue. Live and immunostaining images were superimposed (upper/lower right). Created with BioRender.com.

To assess the stability of *Ugt1a7c* expression in mouse brains across different ages, we employed the UGT1A7C-specific substrate, CDr20, and conducted further evaluation. Interestingly, the fluorescence intensity of CDr20 remained unchanged across all tested ages, ranging from 3 months to 18 months (**Figure 2A**). This indicates that *Ugt1a7c* activity persists throughout the lifespan of the mouse. In addition to physiological condition, treatment with various activators, such as LPS, IFNγ, LPS/IFNγ, ATP, IL-13, or Aβ, did not alter the activity or expression of *Ugt1a7c* in mouse microglia (**Figures 2B, C**). This indicates that the stability of *Ugt1a7c* expression is extensive, even when strong environmental factors or activators are present. The stability of *Ugt1a7c* expression was also confirmed, as all Iba1-positive microglia in an aged AD animal could be labeled with CDr20, irrespective of their localization to Aβ aggregates (**Figure 2D**).

In summary, the expression of particular genes in microglia changes according to their developmental stage, the brain region they reside in, and the surroundings in which they communicate. Microglia express a variety of genes, allowing them to precisely adjust neural circuits and control inflammatory responses. Even though they are only expressed in microglia, there are still genes whose functions and sequences remain unknown. The UGT1A7C is essential for phase II biotransformation reactions, aiding in the elimination of lipophilic substances from the body, and is exclusive to microglia. While the role of *Ugt1a7c* in the brain is still uncertain, its recognition as a microglia marker has been achieved through the development of a fluorescent substrate, despite its low expression level. *Ugt1a7c* remains consistently expressed, regardless of microglial activity, developmental stage, or disease state, making it distinct from other markers (**Figures 1**, **2**).

In recent years, fluorescent probes for functional enzymes have attracted considerable attention because of their inherent advantages, such as high sensitivity, cost-effectiveness, and applicability to high-throughput screening (HTS). However, developing a practical fluorescent probe for a given UGT enzyme remains challenging for the following two reasons. First, UGTs within a subfamily share high amino acid sequence homology (>65%) and usually exhibit broad and overlapping substrate specificity (184). Second, the fluorescence properties of many fluorophores are often “turned off” following O-glucuronidation at the hydroxyl group (116, 189–191). Interestingly, the novel fluorogenic microglia probe, CDr20, identified by unbiased high-content imaging screening with over a thousand of small fluorescent molecules, was a specific exogenous substrate of UGT1A7C after genome-wide CRISPR/Cas9 knockout screening in BV2 microglia. CDr20 was able to label only microglia with high specificity and sensitivity in the mixture of primary glia culture and even in the brain *in vivo* (83). This means that the low expression levels of UGT1A7C in microglia are functional enough for the specific labeling of microglia with its fluorescence substrate.

## Conclusion

Microglial detection with CDr20 is not affected by developmental stages, disease, or environmental factors. This indicates that *Ugt1a7c* is a very stable gene in microglia like housekeeping genes and performs its enzymatic function in both silent and active states of microglia constantly. Although the function of this protein in microglia is not yet fully understood, similar to that of TMEM119, we speculate that this new biomarker is very interesting to other microglia biomarkers because of its unique property as the microglia’s housekeeping gene in the brain and the existence of its specific fluorogenic substrate.

## Data availability statement

The original contributions presented in the study are included in the article/supplementary material. Further inquiries can be directed to the corresponding author.

## Ethics statement

The animal study was approved by Institutional Animal Care and Use Committee (IACUC) of Korea Brain Research Institute (KBRI). The study was conducted in accordance with the local legislation and institutional requirements.

## Author contributions

WK: Conceptualization, Data curation, Software, Validation, Visualization, Writing – original draft, Writing – review & editing. MK: Writing – review & editing. BK: Conceptualization, Data curation, Formal analysis, Funding acquisition, Investigation, Resources, Supervision, Writing – review & editing.
